# 免疫检查点抑制剂相关不良反应预测的研究进展

**DOI:** 10.3779/j.issn.1009-3419.2023.106.20

**Published:** 2023-10-20

**Authors:** Jing ZHANG, Xueqin CHEN, Shenglin MA

**Affiliations:** ^1^310053 杭州，浙江中医药大学第四临床医学院; ^1^Fourth Clinical Medical College, Zhejiang Chinese Medicine University, Hangzhou 310053, China; ^2^310006 杭州，浙江大学医学院附属杭州市肿瘤医院胸部肿瘤科; ^2^Department of Thoracic Oncology, Affiliated Hangzhou Cancer Hospital, Zhejiang University School of Medicine, Hangzhou 310006, China

**Keywords:** 免疫检查点抑制剂, 免疫治疗相关不良反应, 临床特征, 生物标志物, Immune checkpoint inhibitors, Immune-related adverse events, Clinical factors, Biomarkers

## Abstract

免疫检查点抑制剂开启了肿瘤治疗的新纪元，然而免疫治疗在使患者获益的同时也可能引起免疫相关不良反应，这些不良反应会累及全身各器官和系统，并可能发生在治疗的任何阶段，甚至会危及生命。由于临床表现和发病时间的多样化导致对此类不良反应的预测和诊断进一步复杂化。本文旨在对免疫检查点抑制剂相关不良反应的临床特征以及预测的生物标志物进行综述，以帮助临床医生评估药物风险并提前预警不良反应。

## Abstract (en)

The era of tumor treatment has been revolutionized by the advent of immune checkpoint inhibitors. However, while immunotherapy benefits patients, it can also lead to immune-related adverse events that may affect multiple organs and systems throughout the body, potentially even posing a life-threatening risk. The diverse clinical manifestations and onset times of these adverse events further complicate their prediction and diagnosis. The purpose of this paper is to review the clinical characteristics and predicted biomarkers of adverse events related to inhibitors at immune checkpoints, in order to help clinicians evaluate drug risks and early warn adverse events.

免疫治疗已经成为肺癌、黑色素瘤、淋巴瘤、泌尿系等恶性肿瘤的重要治疗手段，其中免疫检查点抑制剂（immune checkpoint inhibitors, ICIs）的出现更是成为恶性肿瘤治疗史上的里程碑事件^[1]^。据统计^[2]^，在美国确诊的转移性癌症中，约43%的患者符合ICIs治疗。然而，由于其靶点和作用机制的特异性，ICIs在解除肿瘤的免疫逃逸时也激活了人体正常组织和器官的免疫系统，从而在相应部位引起自身免疫和炎症反应，称为免疫相关不良事件（immune-related adverse events, irAEs）。irAEs几乎可以发生在全身各大系统，最常见的是皮肤、内分泌、消化和呼吸系统，虽然irAEs大多数病情较轻且具有限制性，但仍有近10%的患者出现严重甚至危及生命的irAEs^[2,3]^。目前对于irAEs的机制尚未十分明确，可能与细胞自身免疫，自身抗体、补体活化，细胞因子及趋化因子释放，遗传学和肠道微生物的改变等有关^[4]^。因此，探索irAEs发生的潜在预测因素和标志物对临床长期安全地使用ICIs具有重要意义。本文就发生irAEs的临床特征以及潜在的生物预测标志物进行综述如下，以求探索其风险因素，为irAEs的诊断和治疗提供重要依据。

## 1 人口学和临床特征

年龄或性别与irAEs的发生未见明显相关性。有研究^[5]^报道接受免疫治疗的实体瘤老年患者，各年龄组irAEs发生率未见显著差异，但≥90岁的患者较90岁以下患者因irAEs所致ICIs停药率更高。但也有少数研究持不同观点，如一项回顾性研究^[6]^分析显示，>65岁ICIs治疗老年患者比25-65岁患者具有更高的irAEs发生风险，这可能是老年患者髓样细胞中c-Jun氨基末端激酶级联反应和含胶原蛋白细胞外基质的基因上调导致。一项包含13项临床研究的荟萃分析^[7]^显示，接受ICIs治疗的癌症患者发生irAEs的性别相关性差异较小，甚至在临床实践中可以不必考虑irAEs管理的性别影响。但irAEs的毒性谱在不同性别中似乎有所不同，女性较男性内分泌系统毒性更为常见，而心血管、神经和皮肤不良反应更少见^[8]^。该现象发生的原因可能是性别导致的激素和免疫反应差异，尤其是女性对自身免疫性疾病的易感性更高，从而导致对自身抗原的免疫耐受性丧失。

日本一项回顾性队列研究^[9]^表明，既往或现在有吸烟史的患者更易发生irAEs，单因素及多因素分析均提示有吸烟史的非小细胞肺癌（non-small cell lung cancer, NSCLC）、胃癌和黑色素瘤患者发生irAEs的概率显著升高。

身体质量指数（body mass index, BMI）的增加已被证实与irAEs的发生呈正相关。在一项关于程序性细胞死亡蛋白1（programmed cell death 1, PD-1）抑制剂治疗恶性黑色素瘤的真实世界研究^[10]^中发现，与BMI≤25.0 kg/m^2^的患者相比，BMI>25.0 kg/m^2^的患者irAEs发生率更高。BMI增加与自身免疫和慢性炎症状态密切相关，肥胖患者常有炎症细胞因子高表达和免疫系统动态重组。

## 2 放疗和慢性肺部疾病

放疗可能为irAEs的易感因素。放疗导致的细胞损伤可引起多种细胞因子释放，并通过多种信号通路直接损伤肺组织，还可募集非特异性免疫细胞间接诱导肺损伤^[11]^。研究^[12]^表明，ICIs治疗的肺癌患者合并放疗，或基线时存在慢性阻塞性肺疾病及肺纤维化等慢性肺部疾病史，发生免疫相关性肺炎的风险更高，但多因素回归分析提示放疗并不是肺部irAEs的独立危险因素。Deng等^[13]^回顾性研究证实，慢性阻塞性肺疾病、既往胸部放疗史和ICIs期间胸部放疗与免疫相关性肺炎事件独立相关，研究者还建立相关风险预测模型，肺气肿、间质性肺疾病、胸腔积液、单药免疫治疗和ICIs合并放疗史可纳入重度肺部AEs风险预测模型。不同部位放疗也可引起不同的irAEs毒性谱，除上述肺部放疗合并免疫治疗引起肺部不良反应外，另有研究^[14]^证实头颅放疗联合ICIs治疗的脑转移瘤患者较单用ICIs的患者irAEs发病率更高，其中以急性中枢神经系统毒性更多见。

## 3 肿瘤瘤种类别

使用同种ICIs治疗不同类型肿瘤的患者抗肿瘤免疫反应不同，因此具有不同的irAEs毒性谱。如在阿替利珠单抗治疗下发生irAEs患者中进行荟萃分析^[15]^，结果表明，免疫相关性肺炎主要见于肺癌患者，甲状腺功能减退和皮疹更常见于晚期肾细胞癌患者。有研究^[16]^分析，288例不同肿瘤瘤种患者中，相较于其他瘤种的患者，黑色素瘤患者更易发生皮肤相关irAEs，NSCLC患者更易发生呼吸系统相关irAEs。

## 4 ICIs类别

irAEs毒性谱、发生率和严重程度因ICIs的类别而异。细胞毒性T淋巴细胞相关蛋白-4（cytotoxic T lymphocyte-associated antigen-4, CTLA-4）抑制剂常见不良反应为结肠炎、垂体炎和皮疹，而PD-1及其程序性死亡配体1（programmed cell death ligand 1, PD-L1）抑制剂患者更常见肺炎和甲状腺功能减退等不良反应。这是由于不同类别的ICIs具有不同的作用靶点。例如CTLA-4在垂体中表达，因此使用CTLA-4单克隆抗体的患者垂体炎发病率更高，而PD-1抑制剂使巨噬细胞激活可导致肺炎发病率增加。研究^[4]^统计，接受CTLA-4抑制剂治疗的患者发生所有级别（1-5级）尤其是高级别（3-5级）irAEs的发病率高于接受PD-1/PD-L1抑制剂治疗的患者。

## 5 血液生物标志物

ICIs可能通过阻断正常组织PD-1和CTLA-4受体直接引起自身免疫，也可产生新自身抗体并使原有自身抗体水平增加，同时使局部促炎细胞因子水平升高，使致病性免疫细胞浸润，引起局部炎症环境，导致器官损伤，这提示炎症标志物及细胞因子等可作为预测irAEs的生物标志物^[2]^。

### 5.1 血细胞

一项ICIs治疗晚期NSCLC的研究^[17]^表明，基线时高水平绝对淋巴细胞计数（absolute lymphocyte count, ALC）患者发生irAEs的风险更大，高水平的基线ALC可能会提高T细胞的潜在抗肿瘤能力及其对免疫治疗的反应能力。另外，中性粒细胞与淋巴细胞比值（neutrophil to lymphocyte ratio, NLR）和血小板与淋巴细胞比值（platelet to lymphocyte ratio, PLR）是全身炎症的生物标志物，都反映了非特异性炎症和免疫反应之间的平衡。有研究^[18]^证实使用ICIs治疗的NSCLC患者基线低水平NLR和低水平PLR与irAEs的发展密切相关。一项ICIs治疗晚期NSCLC的研究^[19]^显示，免疫相关性肺炎患者基线外周血绝对嗜酸性粒细胞计数（absolute eosinophil count, AEC）高于非肺炎AE患者，基线为高水平的AEC与ICIs肺炎风险增加有关。嗜酸性粒细胞具有调节细胞或效应细胞的功能，可通过发挥抗原呈递功能激活T细胞调节各种免疫功能从而引起irAEs。

我们前期的研究^[20]^发现使用ICIs治疗的晚期NSCLC患者，基线外周CD8^+ ^T淋巴细胞与irAEs的发生显著相关，活化的T细胞在杀死肿瘤细胞的同时可能攻击正常的人体组织细胞，形成ICIs相关的毒性。调节性T细胞（regulatory cells, Tregs）和T细胞库在调节自身免疫平衡方面至关重要，ICIs可消耗Tregs，使Tregs功能紊乱，同时调节T细胞库，导致T细胞的自身反应性激活，破坏体内平衡，从而促进新的自身反应性T细胞的发展。因此当Tregs缺乏或重编时将会发生免疫性疾病。在26例已接受抗PD-1的黑色素瘤晚期患者的血样中分离出Tregs细胞，并行转录组测序分析，结果显示Tregs转录组重构与实体瘤irAEs相关，实体瘤irAEs和自身免疫性疾病（autoimmune disease, AID）Tregs转录组基因表达具有一致性，并且实体瘤irAEs和AID存在共表达基因^[21]^。

### 5.2 细胞因子

细胞因子在维持免疫内环境平衡以及参与癌症、感染和自身免疫等病变方面不可或缺，它可以放大促炎和抗炎反应，因此免疫毒性可能与特定的细胞因子有关。接受ICIs治疗的晚期恶性肿瘤患者的前瞻性研究^[22]^表明，发生甲状腺不良反应的恶性肿瘤患者基线白细胞介素（interleukin, IL）-1β、IL-2和粒细胞集落刺激因子（granulocyte colony-stimulating factor, G-CSF）水平较高。这可能是因为这些细胞因子可诱发甲状腺炎，此外IL-2可以促进T细胞增殖和分化，同时活化T细胞，诱导自然杀伤（natural killer, NK）细胞并增强其细胞溶解作用，以此攻击肿瘤细胞及甲状腺细胞。一项回顾性研究^[23]^评估了多种细胞因子与irAEs的相关性，结果显示基线IL-17升高与3级腹泻、结肠炎发病相关，而基线低水平G-CSF也与结肠炎不良反应相关。G-CSF可促进巨噬细胞和调节性T细胞的生成，临床试验表明其对结肠炎具有保护作用，因此基线G-CSF水平下降可能会增加结肠炎AEs的发生风险。此研究还发现基线IL-6、IL-10、血管生成素-1（angiopoietin-1, Ang-1）和CD40配体（CD40 ligand, CD40L）升高与皮肤毒性相关。可能是因为Ang-1有促进微血管增生、破坏屏障稳定和促炎的作用，而CD40L导致自身抗原的表达增强等原因所致。IL-10可由CD4^+ ^T细胞和CD8^+ ^T细胞等多种细胞生成，同时反馈刺激CD8^+ ^T细胞的增殖，并提高T细胞的细胞溶解活性，增加NK细胞的杀伤能力，促进B细胞的存活、增殖和分化。研究^[24]^发现，高水平IL-10与活跃的炎症环境相关，基线IL-10水平较高的患者发生irAEs的风险较高，并且在ICIs治疗期间，IL-10的动态变化仍然与irAEs的发生有关。Tsukamoto等^[25]^在使用PD-L1抑制剂治疗的NSCLC患者中发现趋化因子CXC配体13（chemokine C-X-C motif ligand 13, CXCL13）的增加与irAEs发病率相关。CXCL13是AID的生物标志物，其水平与疾病变化相关，因此，有望通过监测血浆中的CXCL13作为预测irAEs的生物标志物。

### 5.3 血清其他标志物

高乳酸脱氢酶（lactate dehydrogenase, LDH）的患者可能具有较高的全身炎症状态，由此促进更高级别irAEs的发生。研究^[26]^发现免疫相关性心肌炎患者常有较高的基线LDH，随着其水平升高，不良反应的级别也随之升高。因此，临床实践中LDH水平的检测可为分析irAEs的发病率及严重程度提供参考。C反应蛋白（C-reactive protein, CRP）是感染的标志物，癌症晚期的大量组织损伤以及机体组织对肿瘤微环境的免疫反应可增加其表达。研究^[27]^表明使用CTLA-4和PD-1抑制剂患者发生自身免疫性肝炎及甲状腺相关不良反应的基线血清CRP水平显著升高。

### 5.4 蛋白质组学

蛋白质组学可能作为irAEs的预测生物标志物。一项比较白癜风样色素脱失irAEs与蛋白质组学特征的前瞻性队列研究^[28]^结果显示，相较于不良反应阴性患者，白癜风样色素脱失患者具有明显的蛋白质组学特征，表现为外异蛋白A受体（ectodysplasin A receptor, EDAR）上调、淋巴细胞激活基因-3（lymphocyte-activation gene 3, LAG-3）下调和整合素α11（recombinant integrin alpha 11, ITGA11）升高等。有研究^[29]^分析免疫治疗下NSCLC患者初次免疫应答（primary immune response, PIR）测试，结果显示敏感组更不易发生irAEs，并对治疗3周后的患者样本进行分析，发现irAEs阳性组和阴性组存在细胞外基质重塑、补体活化和免疫耐受的蛋白通路显著差异。血清蛋白质组学特征是指与癌症患者免疫反应相关的蛋白质的独特模式。这些蛋白质特征来源于基于质谱的患者血清样本，并可作为生物标志物来预测对免疫治疗的反应。

## 6 自身抗体和AID

自身抗体是由B细胞驱动的体液免疫系统的产物，与AID有关，具有促炎致病作用。有研究^[30]^证实基线自身抗体水平（尤其是抗核抗体和类风湿因子）与irAEs发展相关，但其机制仍然存在争议。自身抗体可引起细胞和组织损伤所致的全身炎症，刺激受体或阻断配体以此抑制靶蛋白功能，并激活补体系统形成免疫复合物沉积引起器官损伤来介导irAEs的发生^[31]^。在一项回顾性分析ICIs治疗黑色素瘤患者的研究^[32]^中发现，基线时自身抗体浓度低，并且从基线到治疗后6周的抗体浓度增大倍数更大的患者会出现更明显的器官特异性irAEs。Daban等^[33]^前瞻性分析了44例ICIs治疗的晚期实体瘤患者预先存在的自身抗体与≥2级irAEs发生之间的关联，发现两者有明显关系，尤其是预先存在的抗甲状腺抗体与相应受体结合导致甲状腺激素分泌异常，由此预测自身免疫性甲状腺功能障碍的发生。此外还有许多自身抗体已被证实与特定不良反应相关，如1型糖尿病抗体阳性的患者较阴性的患者接受治疗后出现糖尿病相关irAEs周期更短^[34]^，神经系统相关不良反应中已发现抗横纹肌抗体可在重症肌无力和肌炎重叠的情况下作为生物标志物^[35]^等。

自身抗体是AID诊断和分类的重要生物标志物，该疾病特征是免疫紊乱导致异常的B细胞和T细胞对机体组织的变态反应^[36]^。一项前瞻性队列研究^[37]^显示，相较于对照患者，AID患者发生全级别、重度和多发性irAEs的风险更大。Pizuorno等^[38]^分析已存在AID的癌症患者在接受ICIs治疗后irAEs的发生率，结果表明桥本甲状腺功能减退症和神经系统AID患者新发irAEs发生率较高。以上结果均提示AID是irAEs发生的潜在易感因素。

## 7 微生物菌群

胃肠道微生物群是指居住在胃肠道中的微生物复杂系统，与宿主建立共生关系并参与宿主健康的稳态过程。目前已知肠道微生物菌群与几类免疫性疾病的发病机制有关，尤其是与炎症性肠病相关的疾病，菌群也被证明可以调节肠道和非肠道irAEs^[39]^。肠道生态失调的特征是微生物菌群多样性减少，导致肠道中部分细菌占主导地位，这可能会影响ICIs的抗肿瘤反应并增加irAEs的发病概率^[40]^。最近一项队列研究^[41]^分析免疫治疗的黑色素瘤患者肠道菌群，结果显示毛螺菌属与链球菌属的特定微生物特征与irAEs相关。另有研究^[42]^拟证实杆菌通过激活或抑制肠道免疫应答来影响结肠炎和皮肤等irAEs的发生，该研究证实拟杆菌与炎症因子有关，可以影响免疫调节和免疫刺激反应的平衡，其富集可减少结肠炎等irAEs，放线菌在胃肠道中建立免疫亚群中起关键作用，也可降低结肠irAEs。

## 8 免疫遗传学

人类白细胞抗原（human leukocyte antigen, HLA）具有高度基因多态性，可将肽配体呈递给T细胞受体，肽结合区域的亲和力改变可影响不同抗原的结合能力，因此HLA被认为是自身免疫的影响因素，也可能在irAEs的发展中发挥作用。研究^[43]^发现特定器官和组织中的irAEs可能与某些HLA多态性有关，如HLA-DRB3*01:01与血小板减少症有关，HLA-DPB1*04:02与低钾血症、白细胞减少和贫血相关，HLA-A*26:01和胆红素升高有关。另有研究^[44]^分析PD-1/PD-L1抑制剂治疗的晚期癌症患者免疫或癌症相关基因中的166个单核苷酸多态性（single nucleotide polymorphism, SNP），其中尿嘧啶N糖基化酶（uracil N glycosylase, UNG）、干扰素ω（interferon omega 1, IFNW1）、干扰素λ4（interferon lambda 4, IFNL4）、PD-L1和CTLA-4与≥3级irAEs相关。

## 9 总结与展望

在免疫治疗时代，ICIs的适应证不断扩大，可伴随而来的irAEs越来越被关注和重视，为提高ICIs疗效并减少毒副作用，使患者能最大程度获益，提早预测irAEs发生，是irAEs全程管理的重点。本文总结描述了有关临床特征、血液生物标志物、肠道微生物群、蛋白组学和免疫遗传学等特征与irAEs发生的相关性，但仍未有精准的标志物可供临床实践使用，因此探索irAEs发病机制、发现新型的预测生物标志物迫在眉睫。此外，综合多个临床因素及生物标志物，建立irAEs预测模型可为安全且精准地使用ICIs提供保障。

**Competing interests**

The authors declare that they have no competing interests.
